# Pannexin 1 regulates bidirectional hippocampal synaptic plasticity in adult mice

**DOI:** 10.3389/fncel.2014.00326

**Published:** 2014-10-15

**Authors:** Alvaro O. Ardiles, Carolina Flores-Muñoz, Gabriela Toro-Ayala, Ana M. Cárdenas, Adrian G. Palacios, Pablo Muñoz, Marco Fuenzalida, Juan C. Sáez, Agustín D. Martínez

**Affiliations:** ^1^Centro Interdisciplinario de Neurociencia de Valparaíso, Facultad de Ciencias, Instituto de Neurociencia, Universidad de ValparaísoValparaíso, Chile; ^2^Escuela de Medicina, Facultad de Medicina, Universidad de ValparaísoValparaíso, Chile; ^3^Centro de Neurobiología y Plasticidad Cerebral, Instituto de Fisiología, Universidad de ValparaísoValparaíso, Chile; ^4^Departamento de Fisiología, Pontificia Universidad Católica de ChileSantiago, Chile

**Keywords:** pannexin 1, hippocampus, LTD, LTP, synaptic plasticity, NMDA receptors, mice

## Abstract

The threshold for bidirectional modification of synaptic plasticity is known to be controlled by several factors, including the balance between protein phosphorylation and dephosphorylation, postsynaptic free Ca^2+^ concentration and NMDA receptor (NMDAR) composition of GluN2 subunits. Pannexin 1 (Panx1), a member of the integral membrane protein family, has been shown to form non-selective channels and to regulate the induction of synaptic plasticity as well as hippocampal-dependent learning. Although Panx1 channels have been suggested to play a role in excitatory long-term potentiation (LTP), it remains unknown whether these channels also modulate long-term depression (LTD) or the balance between both types of synaptic plasticity. To study how Panx1 contributes to excitatory synaptic efficacy, we examined the age-dependent effects of eliminating or blocking Panx1 channels on excitatory synaptic plasticity within the CA1 region of the mouse hippocampus. By using different protocols to induce bidirectional synaptic plasticity, Panx1 channel blockade or lack of Panx1 were found to enhance LTP, whereas both conditions precluded the induction of LTD in adults, but not in young animals. These findings suggest that Panx1 channels restrain the sliding threshold for the induction of synaptic plasticity and underlying brain mechanisms of learning and memory.

## Introduction

Pannexins constitute a family of integral membrane proteins with moderately homologous sequences, but with significant similarity in transmembrane topology when compared to connexins and innexins, which are the classic gap junction channel proteins in vertebrates and invertebrates, respectively (Panchin et al., 2000). The following three members compose the Pannexin family: pannexin 3, which is ubiquitously expressed in many vertebrate tissues, pannexin 1 (Panx1) and pannexin 2, which are abundantly expressed in the brain (Bruzzone et al., 2003). Panx1 has been the most widely studied Pannexin family member thus far and, although originally identified as a gap junction-forming protein, it has been consistently shown to form functional non-junction channels that mediate the exchange of molecules between the cytoplasm and the extracellular space (MacVicar and Thompson, 2010). In fact, Panx1 forms a non-selective channel with high conductance (Bao et al., 2004), through which charged molecules such as ATP have been observed to pass (Bao et al., 2004; Locovei et al., 2006). Nevertheless, permeability to ATP remains controversial (Romanov et al., 2012). It is interesting to note, however, that Panx1 interacts with purinergic receptors mediating the release of ATP towards the extracellular space (Pelegrin and Surprenant, 2006; Locovei et al., 2007). In this regard, mounting evidence has indicated that ATP and purinergic signaling are involved in diverse functions in the central nervous system (CNS), such as neurotransmission, neuromodulation and inflammation (Fields and Stevens, 2000). In this sense, Panx1 channels could also be important contributors to such processes (Silverman et al., 2009; Prochnow et al., 2012).

In the CNS, Panx1 mRNA is expressed in a development-dependent manner in the cerebellum, cerebral cortex, olfactory bulb and hippocampus, exhibiting high levels in embryonic and young systems, and declining in adult tissue (Vogt et al., 2005). At the cellular level, Panx1 channels have been identified in astrocytes, pyramidal cells and interneurons (Vogt et al., 2005; Huang et al., 2007; Zoidl et al., 2007). In neurons, Panx1 channels have been detected in postsynaptic densities, where they co-localize with glutamate receptors and scaffold proteins such as postsynaptic density protein 95 (PSD-95; Zoidl et al., 2007). However, the functional significance of the postsynaptic localization of Panx1 channels is not completely understood. The absence of Panx1 was recently shown to increase long-term potentiation (LTP) along with behavioral alterations, including increases in anxiety as well as spatial and recognition impairments, suggesting that Panx1 modulates synaptic plasticity and is needed for proper learning (Prochnow et al., 2012). In the present report, we extended these studies to other forms of synaptic plasticity. We found that blockade of Panx1 channels or lack of Panx1 causes a shift from long-term depression (LTD) to LTP. These results suggest that Panx1 channels could regulate the sliding threshold for excitatory synaptic plasticity, hence increasing the likelihood of synaptic strengthening elicited by conditioning stimulation.

## Materials and methods

### Animals

The experiments were carried out in 1- to 12 month-old male C57BL/6 or Panx1^−/−^ mice. The generation of Panx1^−/−^ (KO) mice has been described previously (Anselmi et al., 2008). Mice were housed at 21 ± 1°C at constant humidity (55%) and in a 12/12 h dark–light cycle, with a light phase from 08:00 AM to 08:00 PM. Food and water were provided *ad libitum*. The use and care of the animals were approved by the Ethics and Animal Care Committee of Universidad de Valparaíso.

### Electrophysiology

Hippocampal slices were prepared as previously reported (Ardiles et al., 2012). Animals were sacrificed under deep halothane anesthesia, and their brains were quickly removed. The hippocampus was removed and sectioned into 300–400 µm thick slices by using a vibratome (Vibratome 1000 plus, Ted Pella Inc., CA, USA). The slices were transferred and maintained for 1 h at room temperature in normal artificial cerebrospinal fluid (ACSF). Normal ACSF was similar to the dissection buffer, except that sucrose was replaced by 124 mM NaCl, MgCl_2_ was lowered to 1 mM, and CaCl_2_ was raised to 2 mM. All recordings were collected in a submersion-recording chamber perfused with ACSF (30 ± 0.5°C; 2 ml/min). Field excitatory postsynaptic potentials (FPs) were evoked by stimulating Schaffer collaterals with 0.2 ms pulses delivered through concentric bipolar stimulating electrodes (FHC), and were recorded extracellularly in CA1 stratum radiatum. Baseline responses were recorded by using half-maximum stimulation intensity at 0.033 Hz. Basal synaptic transmission was assayed by determining input–output relationships from FPs generated by gradually increasing the stimulus intensity; the input was the peak amplitude of the fiber volley (FV), and the output was the initial slope of FP. Paired-pulse facilitation was elicited by using an interstimulus interval range between 50–500 ms. Long-term potentiation was induced by high frequency stimulation (HFS; Tang et al., 1999) consisting of a single tetanus of 100 Hz or by theta burst stimulation (TBS; Ardiles et al., 2012), consisting of four theta epochs composed by 10 trains of four pulses (at 100 Hz) delivered at 5 Hz. Long-term depression was induced by low frequency stimulation (LFS; Ardiles et al., 2012) or by paired-pulse low frequency stimulation (PP-LFS; Ardiles et al., 2012), both consisting of 900 pulses at 1 Hz. Long-term depression also was induced by 900 pulses at 5 and 10 Hz (Cui et al., 2013). These protocols were delivered following 20 min of baseline transmission. For chemical-LTD, slices were superfused for 5 min with 30 µM NMDA. Panx1 channels were blocked with 200 µM probenecid. Glial glutamate transporter was blocked with 10 µM DL-TBOA. D-AP5, NMDA and DL-TBOA were obtained from Tocris (Bristol, UK). Probenecid was obtained from Molecular Probes (Eugene, OR, USA).

### Immunoblotting

After electrophysiological recordings, hippocampal slices were quickly frozen and processed for Western blotting as previously described (Ardiles et al., 2012). Samples were homogenized in ice-cold lysis buffer (150 mM NaCl, 10 mM Tris-Cl, pH 7.4, EDTA 2 mM, 1% Triton X-100 and 0.1% SDS), supplemented with a protease and phosphatase inhibitor cocktail (Thermo Scientific, Rockford, IL, USA) by using a homogenizator. Protein samples were centrifuged twice for 5 min at 14,000 revolutions per minute (rpm) (4°C). Protein concentration was determined with the BCA Protein Assay Kit (Thermo Scientific, Rockford, IL, USA). For synaptic proteins, 40 µg of protein per lane were resolved by 10% SDS-PAGE, followed by immunoblotting on PVDF membranes (Merck Millipore, Merck KGaA, Darmstadt, Germany) with mouse anti-β-actin (Santa Cruz Biotechnology, Dallas, TX, USA) and rabbit anti-Panx1 (CT-395; Penuela et al., 2007). Band intensities were visualized by enhanced chemiluminiscence kit (ECL, Pierce, Thermo Scientific, Rockford, IL, USA) and the intensity of each band was scanned and densitometrically quantified through the use of Image J software (NIH, Bethesda, MD, USA).

### Biotinylation

Surface biotinylation was performed in acute hippocampal slices as previously reported with minor modifications (Lee et al., 2003; Thomas-Crusells et al., 2003). Slices were briefly preincubated in ACSF at 30°C for 1 h, washed twice with ice-cold ACSF and then incubated with sulfo-NHS-SS-Biotin (Thermo Scientific; 1 mg/ml in ACSF) for 45 min on ice with gentle rotation. Excess biotin was removed by means of two brief washes with 10 mM lysine (in ACSF) and two ACSF washes. Slices were then lysed in 500 µl of lysis buffer, centrifuged at 14,000 rpm for 5 min at 4°C and supernatants were discarded. Pellets were resuspended in lysis buffer and biotinylated cell-surface proteins were precipitated with high capacity neutravidin agarose resin (Thermo Scientific, Rockford, IL, USA) and the mixture was rotated overnight at 4°C. After several washes with lysis buffer, precipitates were collected by centrifugation (14,000 rpm for 1 min) and detected by immunoblot.

### Quantitative reverse transcription polymerase chain reaction

Total RNA was extracted from brain tissue by using the SV Total RNA Isolation System (Promega Corp., Madison, WI, USA) according to the manufacturers’ instructions. RNA concentration was determined spectrophotometrically at 260 nm. One microgram of RNA was incubated with 0.5 µg of oligo-dT primer and reverse transcriptase (ImProm-II Reverse Transcription; Promega), according to the manufacturer’s specifications. Levels of Panx1 RNA were analyzed by qRT-PCR using the following primers: Forward 5′-GCCAGAGAGTGGAGTTCAAAGA-3′; Reverse 5′-CATTAGCAGGACGGATTCAGAA-3′. qRT-PCRs were performed in a total volume of 20 µl consisting of 100 ng of cDNA, 10 µl of Kapa Sybr^®^ Fast Master mix (Kapa Biosystems) and 0.4 µl of each primer under the following cycling conditions: uracil DNA glycosylase (UDG) activation at 50°C for 2 min, polymerase activation at 95°C for 5 min, followed by 40 cycles of denaturation at 95°C for 10 s, annealing at 55°C for 30 s, extension at 72°C for 30 s, followed by a melt curve of 95°C for 15 s, 55°C for 15 s and 95°C for 15 s. Equal loading of cDNA was confirmed by amplification of the cyclophilin with the following primers: Forward 5′- AGGTCCTGGCATCTTGTCCAT-3′ and Reverse 5′-GAACCGTTTGTGTTTGGTCCA-3′. The purity of the amplified products was checked with a dissociation curve.

### Statistics

All data are presented as mean ± standard error of the mean (SEM). Data analysis was carried out using the Prism software (GraphPad Software Inc.). Statistical comparisons were performed by means of a two-tailed t-test and one-way ANOVA, followed by a Tukey’s or repeated measures/two-way ANOVA and subsequently by Bonferroni’s *post hoc* tests.

## Results

### Panx1 RNA and protein levels decrease in the brain with age

**Figure 1:**
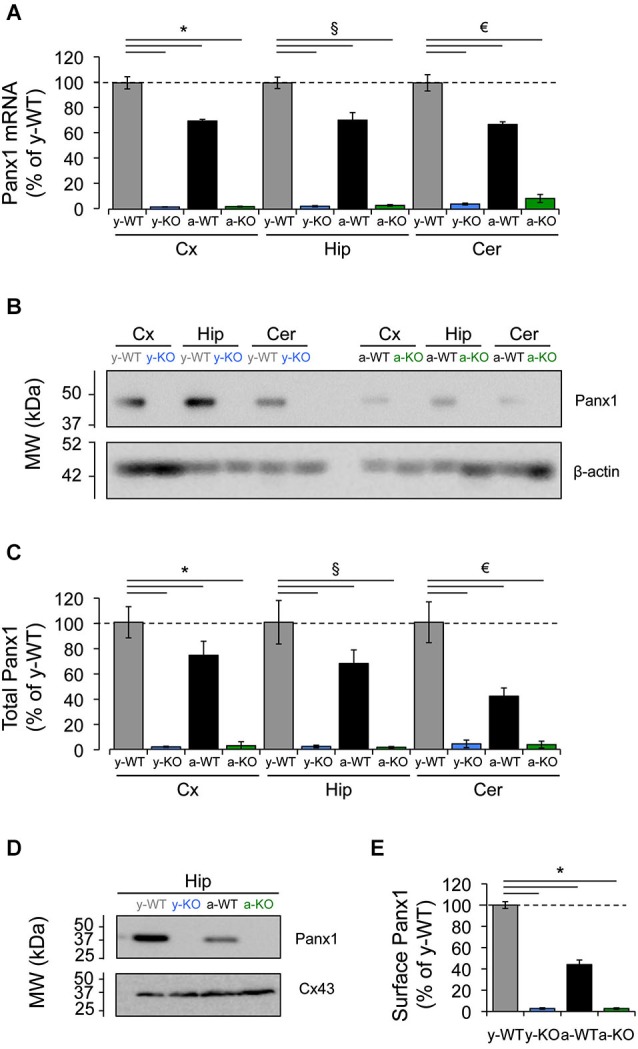
**Decreased expression of Panx1 transcript and protein in the adult mice brain. (A)** Quantitative RT-PCR analysis of relative abundance of Panx1 mRNA in the cerebral cortex (Cx), hippocampus (Hip), and cerebellum (Cer) from young wild type (y-WT, gray), young Panx1 knockout (y-KO, blue), adult wild type (a-WT, black) and adult Panx1 knockout mice (a-KO, green). The transcript values were normalized to the levels of cyclophilin (Cyp1) (*N* = 4). **(B)** Representative Western blot showing the expression levels of Panx1 protein in homogenates of cerebral cortex (Cx), hippocampus (Hip) and cerebellum (Cer) of young or adult animals. β-actin expression in samples was used for loading control. **(C)** Quantification of Panx1 protein expression by densitometry analysis of bands from four independent Western blots (*N* = 4), including the one shown in **(B)**. Values were normalized to β-actin loading control. **(D)** Western blots of plasma membrane biotinylated proteins of hippocampal slices probed with anti-Panx1 and anti-Cx43 antibodies. **(E)** Quantification of protein expression by densitometry analysis of Panx1 bands from three independent Western blots (*N* = 3) like the one shown in **(D)**. All data are plotted as mean ± SEM related to results of y-WT animals. Statistical differences were calculated using 2 way-ANOVA, followed by *post hoc* Bonferroni’s test. * *p* < 0.05 vs. y-WT Cx; § *p* < 0.05 vs. y-WT Hip; € *p* < 0.05 vs. y-WT Cer.

Early expression analyses have revealed a widespread distribution of Panx1 mRNA in many areas of the CNS, including the olfactory bulb, cerebral cortex, hippocampus and cerebellum (Bruzzone et al., 2003), exhibiting changes that occur in a time-dependent manner that is, Panx1 mRNA is expressed at high levels in embryonic and young postnatal whole brain, but declines during adulthood (Vogt et al., 2005). Accordingly, quantitative-RT-PCR analyses of samples from different brain areas revealed a decrease in Panx1 mRNA levels for adult (9–12 months) wild type (WT) mice compared to young (1 month) WT mice (Figure 1A). Consistent with this, we detected almost similar levels of reduction in total Panx1 protein levels in the adult cerebral cortex, hippocampus and cerebellum compared to Panx1 levels found in the same brain areas of young WT animals (Figure 1C). Because Panx1 channels are localized mainly in the plasma membrane where they exert their main functions, we decided to investigate whether the reduction in total Panx1 protein levels in WT adult mice would also result in similar levels of reduction in the membrane compartment. We observed even larger levels of reduction in protein Panx1 levels in the plasma membrane of adult hippocampus tissue compared to young (about 60% of reduction; Figures 1D,E). As a control for the process of biotinylation of plasma membrane proteins we analyzed the levels of biotinylation of Connexin43 (Cx43), which is another membrane protein that form hemichannels mainly in the astroglial cells (Dermietzel et al., 1989). We observed that Cx43 levels were slightly increased during adulthood (Figures 1D,E), consistent with the importance of this proteins in astroglial function. These results suggest that reduction in the plasma membrane levels of Panx1 was a specific process not affected by the biotinylation procedure. Finally, both Panx1 mRNA and protein were absent in Panx1^−/−^ (KO) mice (Figure 1). These data confirm previously reported age-dependent expressions of Panx1 in the CNS and encourages us to evaluate whether Panx1 channels play a role in synaptic functions and how they may be affected by age.

### Panx1 channels regulate excitatory synaptic transmission in adult but not in young brain

**Figure 2:**
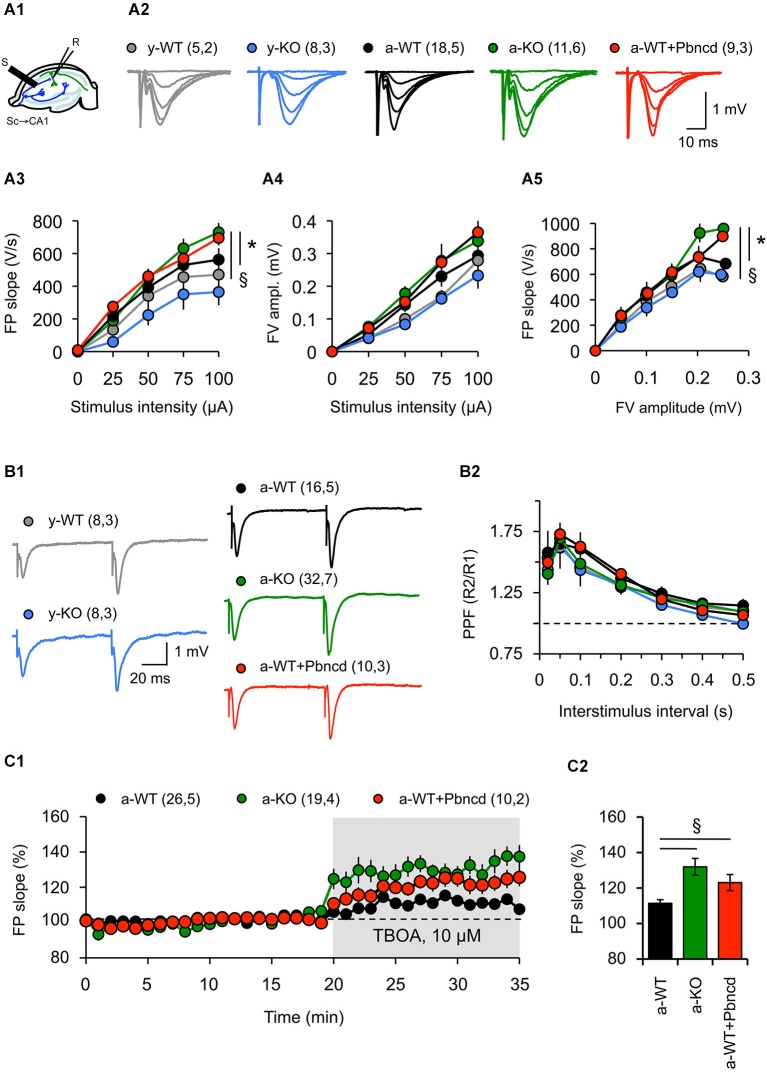
**Absence and blockade of Panx1 channels alter hippocampal synaptic transmission in adult, but not in young mice. (A1)** Cartoon depicting stimulus-record electrode configuration to record synaptic activity in Sc-CA1 synapse in hippocampal slices. **(A2)** representative FP at different stimulus intensities for young wild type (y-WT, gray line), young Panx1 knockout (y-KO, blue line), adult wild type (a-WT, black line), adult wild type plus probenecid 100 µM (a-WT+Pbncd, dotted line) and adult Panx1 knockout mice (a-KO, green line). **(A3–A5)** Input-output curves showing the relationship between FP slope **(A3)**, fiber volley amplitude **(A4)** and stimulus intensity; and fiber volley amplitude and FP slope **(A5)**. An increased FP slope was observed in a-WT+Pbncd and a-KO mice compared to either y-WT or a-WT mice. **(B1)** Representative FP traces at interstimulus intervals of 100 ms. **(B2)** Paired-pulse facilitation (PPF) of the FP at various interstimulus intervals. No significant differences were observed between WT and KO mice. **(C1)** Absence and blockade of Panx1 channels significantly increased glutamate release and spillover in adult animals. DL-TBOA (TBOA 10, µM) was perfused after 20 min of basal transmission. **(C2)** Averaged increments in basal synaptic transmission induced by TBOA. The values in parentheses indicate the number of hippocampal slices (left) and the number of animals (right) used. All data are plotted as mean ± SEM. Statistical differences were calculated using ANOVA, followed by *post hoc* Bonferroni’s test. Asterisks indicate statistical significance of the observed differences. *p < 0.05 vs. y-WT; § *p* < 0.05 vs. a-WT.

There is some evidence for the participation of Panx1 channels in synaptic activity. For example, prolonged activation of Panx1 channels triggered by increased NMDAR activity leads to aberrant ionic currents and abnormal neuronal bursting, which contribute to epileptiform activity (Thompson et al., 2008). In line with the latter, lack of Panx1 improves the outcome of kainic acid-induced status epilepticus in juvenile mice (Santiago et al., 2011), whereas both Panx1 blockade and the P2X_7_ receptor silencing increase susceptibility to pilocarpine-induced seizures in adult mice (Kim and Kang, 2011). Moreover, the absence of Panx1 leads to increased excitability in the adult hippocampus (Prochnow et al., 2012). In order to evaluate the role of Panx1 channels in synaptic transmission at different ages, we recorded synaptic activity in Schaffer collateral-CA1 synapses (Sc-CA1) evoking FP (Figure 2A). We noted in young animals (1 month) that FP slopes and FV amplitudes evoked by different stimulation intensities were indistinguishable between slices from young WT (y-WT) and KO (y-KO) mice, suggesting that lack of Panx1 does not affect excitability (Figures 2A3,A4). Consequently, input-output curves showed no difference in basal transmission between young transgenic mice and WT littermates (Figure 2A5). However, we found a significant increase in FP slopes in slices obtained from adult KO mice (a-KO) without significant changes in FV amplitude for high stimulation intensities compared to slices of adult WT (a-WT, Figures 2A3, 4). Similar results were obtained in slices from a-WT incubated with the Panx1 channel blocker probenecid (a-WT+Pbncd) as compared to untreated slices (Figures 2A3,A4). Input-output curves revealed small but statistically significant differences between a-WT+Pbncd and a-KO compared to a-WT mice, showing that when the same number of axons was recruited after stimulation (same FV value) there was a slightly larger FP in slices from a-WT+Pbncd and a-KO mice (Figure 2A5). These results suggest that the elimination of Panx1 signaling increases the release of transmitters, or increases postsynaptic responsiveness. To explore the first possibility, we measured paired pulse facilitation (PPF) as a function of presynaptic activity and found no differences between the groups (Figure 2B). Although these results indicate that the absence of Panx1 does not affect the release probability, we cannot rule out that Panx1 channels contribute to presynaptic functions. In fact, it has been suggested that the absence of Panx1 could reduce ATP catabolism and increase extracellular adenosine levels, therefore increasing glutamate release (Prochnow et al., 2012). Consequently, in slices incubated with a glial glutamate transporter blocker (TBOA, 10 µM) to increase glutamate availability at synapse, we observed larger FP slopes in slices from a-KO and a-WT+Pbncd compared to slices from a-WT (Figures 2C1,C2). This indirectly suggests that Panx1 channels could contribute to controlling the probability of glutamate release. However, whether the blockade or absence of Panx1 affects postsynaptic mechanisms remains to be determined.

**Figure 3:**
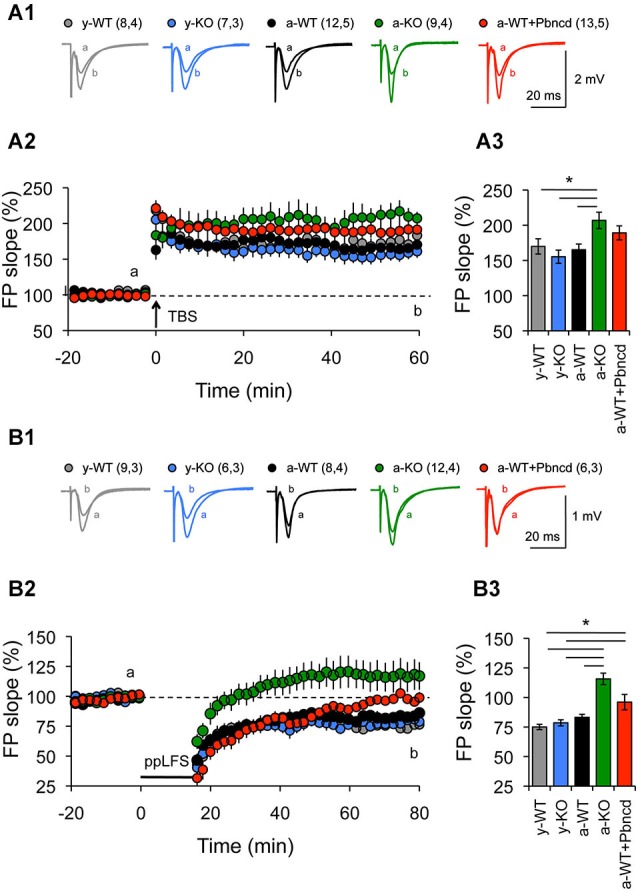
**Increased long term potentiation (LTP) and absent long term depression (LTD) in the Schaffer collateral–CA1 pathway from adult Panx1 knock-out and WT mice treated with Pbncd. (A1)** Representative traces of FPs recorded 1 min before (a) and 60 min after (b) TBS. **(A2)** LTP obtained in slices from young wild type (y-WT, gray line), young Panx1 knockout (y-KO, blue line), adult wild type (a-WT, black line), adult wild type plus probenecid 100 µM (a-WT+Pbncd, red line) and adult Panx1 knockout mice (a-KO, green). Long-term potentiation was induced by the delivery of TBS at the time indicated by the arrow. **(A3)** Magnitude average of LTP determined as responses between 50 and 60 min after TBS. Long-term potentiation was significantly different for a-KO mice compared to y-WT, y-KO and a-WT mice. **(B1)** Representative traces of FPs recorded 1 min before (a) and 60 min after (b) PP-LFS. **(B2)** Long-term depression obtained in slices from y-WT (gray line), y-KO (blue line), a-WT (black line), a-WT plus probenecid 100 µM (a-WT+Pbncd, dotted line) and a-KO hippocampal slices (green line). Long-term depression was induced by the delivery of PP-LFS at the time indicated by the line. **(B3)** Magnitude average of LTD determined as responses between 50 and 60 min after PP-LFS. Long-term depression was absent in a-WT+Pbncd and in a-KO mice. The values in parentheses indicate the number of hippocampal slices (left) and the number of animals (right) used. All data are plotted as mean ± SEM. Statistical differences were calculated using ANOVA, followed by *post hoc* Tukey test. Asterisks indicate statistical significance of the observed differences (*p < 0.05).

**Figure 4:**
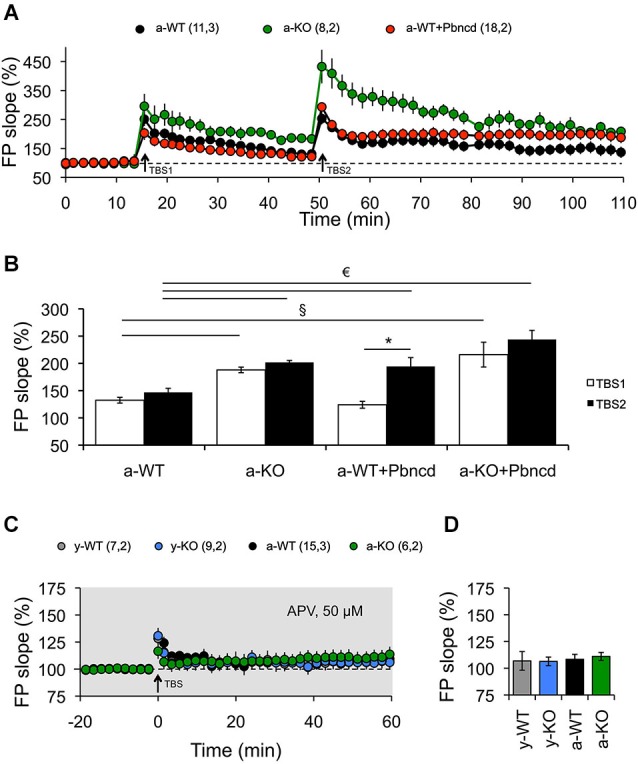
**Blockade or absence of Panx1 channels facilitates the induction of LTP. (A)** Long-term potentiation induced by one TBS (TBS_1_). TBS1 induced a stable LTP in adult Panx1 knockout (a-KO, green square), but transient LTP in adult wild type (a-WT, black circle), which returned to baseline after 30 min. A second TBS (TBS2) applied to the same synapses elicited significantly more potentiation in a-KO but not in a-WT mice. The blockade of Panx1 with Pbncd added 20 min before the application of the TBS2, induced a significant enhancement of potentiation in a-WT (a-WT+Pbncd, red circle). **(B)** Magnitude averages of LTP were determined as responses between 30 and 35 min after the first TBS (open bar) and 50 and 60 min after the second TBS (filled bar). **(C)** Long-term potentiation induced by TBS was completely blocked by the incubation of APV 50 µM. **(D)** Magnitude average of LTP determined as responses between 50 and 60 min after TBS. The values in parentheses indicate the number of hippocampal slices (left) and the number of animals (right) used. *p < 0.05 for TBS1 vs. TBS2; § *p* < 0.05 between TBS1; € *p* < 0.05 between TBS2. All data are plotted as mean ± SEM.

### Blockade or lack of Panx1 channels facilitates LTP and obliterates LTD only in adult animals

It is believed that memories are stored by modifications in synaptic connections between neurons. Two types of activity-dependent synaptic modifications are LTP and LTD, which have received considerable support to be considered as cellular memory mechanisms (Bliss and Collingridge, 1993). In the hippocampus, both LTP and LTD can be elicited at the same synapses by different frequencies of stimulation, where high frequencies induce LTP and low frequencies induce LTD (Dudek and Bear, 1992, 1993). The most accepted model states that high and low frequencies of presynaptic stimulation evoke different levels of postsynaptic calcium influx through an NMDAR-dependent pathway leading to the activation of phosphatases or kinases, respectively (Lisman, 1989; Lüscher and Malenka, 2012). As suggested by Prochnow et al. (2012), Panx1 stabilizes synaptic plasticity and is needed for learning. However, this study was restricted to the role of Panx1 in LTP in adult mice, without considering the analysis of other types of synaptic plasticity. Hence, we decided to evaluate the role of Panx1 channels in bidirectional synaptic plasticity with age by using different protocols and frequency stimulations to induce LTP and LTD in the Sc-CA1 pathway.

**Figure 5:**
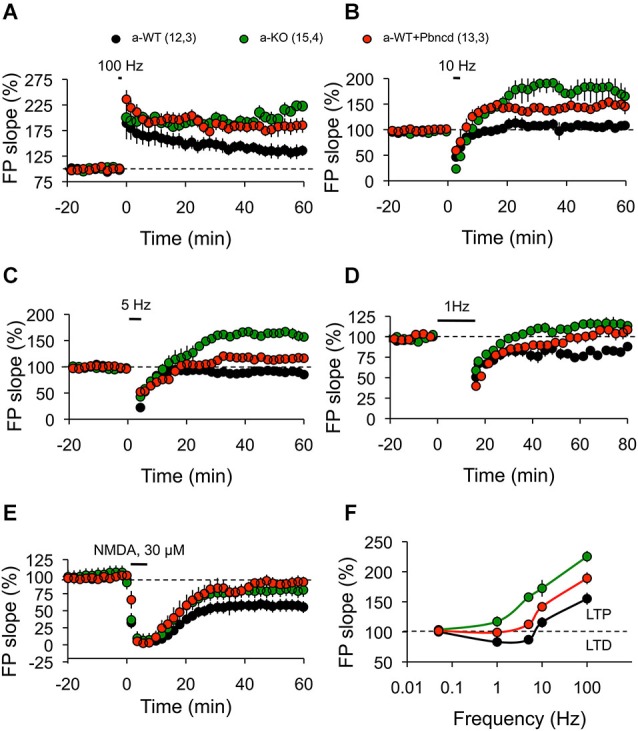
**Absence or blockade of Panx1 channels modifies the threshold for inducing excitatory hippocampal synaptic plasticity. (A)** One tetanus (100 Hz, 1 s) induced significantly more potentiation in adult wild type plus Pbncd (a-WT+Pbncd, white circle) and adult Panx1 knockout (a-KO, green circle) compared to adult wild type (a-WT, black) mice. **(B)** 10 Hz stimulation for 1.5 min evoked significant potentiation in a-WT+Pbncd and a-KO mice compared to a-WT mice. **(C)** 5 Hz stimulation for 3 min produced potentiation in a-WT+Pbncd and a-KO mice, whereas it produced a slight depression in a-WT. **(D)** 1 Hz stimulation for 15 min induced a reliable LTD in WT mice, whereas it elicited a mild potentiation in a-WT+Pbncd and a-KO mice. **(E)** 30 µM of NMDA for 5 min induced LTD in all groups. **(F)** Summary of data for synaptic plasticity at different frequencies of stimulation. The values in parentheses indicate the number of hippocampal slices (left) and the number of animals (right) used. All data are plotted as mean ± SEM.

First, we used two protocols that we have used before to reveal age-dependent changes in LTP and LTD (Ardiles et al., 2012). We observed that strong conditioning stimulation (4TBS; 4 × 100 Hz) elicited potentiation in both young and adult animals. However, significantly enhanced LTP was exhibited by a-KO compared with the other groups (Figure 3A). On the other hand, reliable LTD was elicited with a PP-LFS protocol (1 Hz, 15 min) in brain slices of both y- and a-WT and in brain slices from y-KO mice (Figure 3B). However, this stimulation resulted in transient LTD in a-WT+Pbncd that decayed back to baseline after an hour. Long-term depression however, was absolutely absent in a-KO mice, where this protocol elicited an enduring potentiation (Figure 3B). These data suggest that lack of Panx1 impairs NMDAR-dependent plasticity in adults but not in young animals. To further confirm the possible effects of Panx1 blockade on LTP, we used a stimulation protocol that normally induces transient and non-sustained LTP (Figure 4). We observed that one TBS (1 × 100 Hz; TBS1) protocol induced transient LTP in WT slices, which returned to baseline levels after 20–30 min of applying the protocol (Figure 4A). Interestingly, the same conditioning stimulus (TBS1) produced significantly greater potentiation levels in slices from KO mice, which remained stable after 20–30 min compared to WT slices (Figures 4A,B). When a second TBS (TBS2) was delivered 35 min after the application of the first TBS (TBS1), we observed an increase in the synaptic response in both WT and KO slices. However, this potentiation was maintained for a longer time only in KO slices, while it fell close to baseline levels in WT slices (Figure 4A). In the presence of probenecid, which was added 20 min before the application of TBS2, we observed a robust potentiation in WT slices, reaching similar levels than those observed in KO slices, which remained stable for an hour after TBS2 application (Figure 4B). To rule out non-specific effects of probenecid and/or the participation of other Pannexins expressed in neurons, such as Panx2, slices from KO animals were incubated with probenecid to perform similar experiments as those described above. We found that probenecid did not modify LTP magnitudes compared to untreated KO slices (Figure 4B). These results suggest that probenecid affects LTP only in WT animals, implying that its effects on LTP were specific over Panx1 channels. Consistent with the results described thus far using preconditioning stimuli, we observed that a high frequency stimulation protocol (100 Hz, 1 s; Figure 5A), which is a standard protocol to induce LTP, overall reiterates the same results and clearly shows that the elimination of Panx1 or its blockage potentiates LTP independently of the protocol used to induce it. These protocols induced NMDAR-dependent LTP, since the increase of LTP was completely blocked by APV (Figures 4C,D). These findings suggest that Panx1 could constrain the synaptic strengthening induced by conditioning stimulation, and when Panx1 is blocked or absent, a change in the threshold of synaptic plasticity occurs in adult animals.

To further test this prediction, we then investigated how synaptic plasticity induced by different frequencies of stimulation is affected by blockage or deletion of Panx1. Reliable LTD was elicited with a standard LFS protocol (900 pulses, 1 Hz) in slices from a-WT animals, but it was absent in a-KO animals (Figure 5D). Long-term depression was also significantly reduced in a-WT+Pbncd, in which LTD decayed quickly back to baseline after an hour (Figure 5D). This absence of LTD observed in a-KO and in a-WT+Pbncd was also evident when using other frequencies of stimulation (5–10 Hz) to induce LTD (Figure 5C). On the contrary, the protocols of 900 pulses at 10 Hz (Figure 5B) and 5 Hz (Figure 5C) to induce LTD in a-KO slices evoked a prolonged form of potentiation instead of depression (Figures 5B,C). Despite the absence of LTD elicited with conditioning stimulation, the application of NMDA (30 µM during 5 min) induced chemical-LTD in slices from all animals groups, although a-WT showed a slightly, but not significantly greater depression of the synaptic responses compared to a-WT+Pbncd and a-KO (Figure 5E), thus indicating that the lack or blockade of Panx1 also affects the induction of this form of LTD.

## Discussion

Recent studies have highlighted the participation of Panx1 in LTP, suggesting that Panx1 is involved in this mechanism of synaptic plasticity (Prochnow et al., 2012). Here, we have tested the hypothesis that the blockade of Panx1 not only affects LTP, but also alters LTD in the hippocampus in an age-dependent manner. Our results suggest that the blockade or the lack of Panx1 modifies the threshold for the induction of these forms of bidirectional synaptic plasticity only in adult animals.

First, we confirmed that Panx1 transcripts and protein are expressed in different brain areas, including the hippocampus of young and adult animals. However, contrary to a previous study where an age-dependent decrease in Panx1 expression was observed by *in situ* hybridization (Vogt et al., 2005), we found only a slight reduction in total Panx1 protein levels in adults compared to young animals. Interestingly, by comparing the plasma membrane fractions (Figure 1E) we observed that the adult hippocampus expresses less than 40% of the amount of Panx1 expressed in the young animals. These results strongly suggest that there is an important cellular re-distribution of Panx1 in adult animals. Indeed, multiple subcellular distributions of Panx1 have been recently reported for principal neurons in the hippocampus, cerebellum, olfactory bulb and thalamus by using different antibodies (Cone et al., 2013). However, reported differences in immuno-staining patterns observed with imaging (Cone et al., 2013) suggest important cross-reactions of the commercially available antibodies, which make it difficult to evaluate Panx1 expression by immunolocalization.

Second, we found that upon high intensity stimulation, synaptic transmission in the adult hippocampus increases in conditions where Panx1 is blocked or absent (Figure 2), indicating that Panx1 regulates excitatory synaptic transmission. Moreover, our findings that the blockage of the glial glutamate transporter significantly increases excitatory transmission in both a-KO mice and a-WT+Pbncd support a previously suggested mechanism, in which Panx1 modulates a presynaptic component of the synaptic transmission by increasing glutamate release and spillover (Prochnow et al., 2012). However, since we observed a greater FP slope in a-KO and a-WT+Pbncd animals compared to a-WT mice (Figure 2A), we cannot rule out a postsynaptic contribution, for example, in the mobilization of glutamate receptors.

Third, in agreement with a previous report (Prochnow et al., 2012) we observed increased LTP in the Schaffer Collateral–CA1 pathway of a-KO and a-WT+Pbncd animals (Figure 3A), whereas prolonged LTP was observed in a-KO slices induced with protocols that in WT mice elicited LTD (Figure 3B). This seems to indicate that a metaplastic shift and therefore a modification in the threshold for synaptic plasticity would occur. In fact, a shift in the LTP/LTD induction threshold produced by the blockage or lack of Panx1 was evident after plotting the responses to the different frequency stimulations. In this regard, a moderate and strong shift to the right in the frequency-response curve was observed for a-KO and a-WT+Pbncd slices, respectively (Figure 5F). These effects were restricted to adult animals in agreement with the idea that different synaptic plasticity mechanisms could operate in young and adult animals (Foster, 1999).

**Figure 6:**
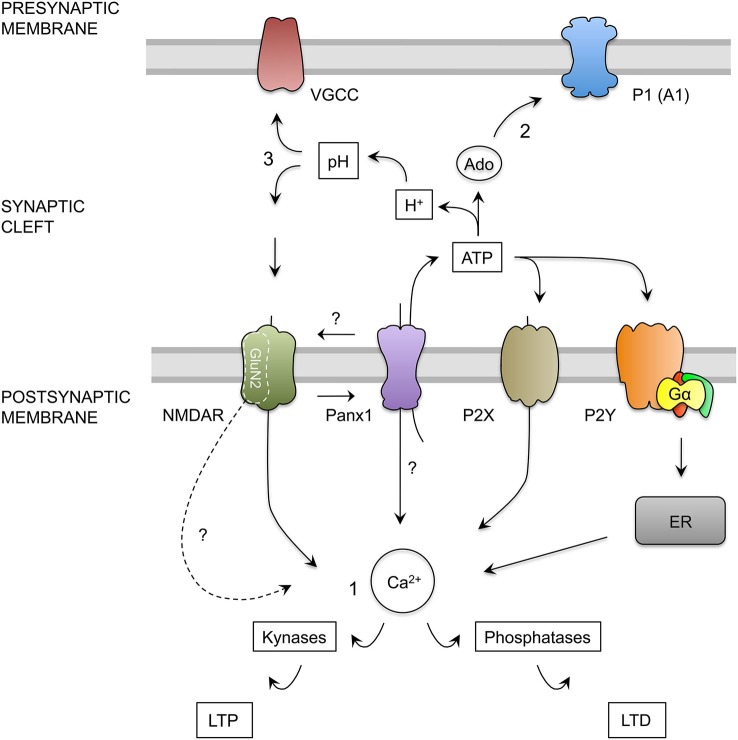
**Hypothesis for the role of Panx1 channels in the regulation of excitatory synaptic plasticity. (1)** Calcium Hypothesis. Upon neuronal activity, NMDAR activation trigger calcium influx into dendritic spine. Depending on the kinetics and magnitude of the calcium concentration increments, kinases or phosphatases are activated promoting the insertion or remotion of AMPARs mediating LTP and LTD respectively (Lüscher and Malenka, 2012). NMDAR activation, probably through depolarization of post-synaptic membrane, activates Panx1 channels (Thompson et al., 2008) producing more calcium influx and ATP release by Panx1 channels. ATP released by Panx1 channels may activate ionotropic (P2X) and metabotropic (P2Y) purinergic receptors. P2X depolarizes the membrane and allows calcium entry, whereas P2Y controls calcium release from intracellular stores such as endoplasmic reticulum (ER) through the activation of G protein (Gα), therefore both receptors may contribute to the increase in the cytosolic calcium concentration (Collo et al., 1996; Yamazaki et al., 2003; Wang et al., 2004; Abbracchio et al., 2009). In addition, Panx1 channels could interact with specific NMDAR subunits exerting a modulatory effect over these receptors. We also speculate that Panx1 channels could facilitate the localization or function of specific NMDAR subunit in the post-synaptic membrane, therefore in the absence of Panx1, a greater activation of kynases instead phosphatases could be due to a change in the composition of GluN2 subunits, leading a change in the kinetics and calcium influx through NMDARs. **(2)** Activation of pre-synaptic Adenosine receptors Hypothesis. In the synapses, ATP release through Panx1 channels could be converted into adenosine (Ado), which in turn can activate P1 purinergic receptors (adenosine receptors). The activation of A1 adenosine receptor (A1), located in the presynaptic terminals reduces the release of glutamate. Therefore, in the absence of Panx1 the depletion of extracellular ATP and adenosine promotes an increase in the neurotransmitter release (Prochnow et al., 2012). **(3)** Regulation of synaptic cleft pH Hypothesis. Extracellular ATP hydrolysis also generates protons and phosphate that produce a decrease in the extracellular pH (Vroman et al., 2014). This acidification could inhibit both, voltage gated calcium channels (VGCC) present in the presynaptic terminal, and NMDAR on postsynaptic membrane, reducing glutamate release and NMDA receptor activation. The absence of Panx1 channels or their inhibition prevents the release of ATP, stops the production of phosphate buffer and produce the alkalization of the synaptic cleft, which increase the release probability and the activation of NMDARs.

It has been well established that aging is associated to a shift in synaptic plasticity, favoring a decrease in synaptic transmission and in the ability to induce LTP (reviewed in Kumar, 2011). These changes are already observed in middle-aged rodents (Rex et al., 2005; Fouquet et al., 2011). Nevertheless, we did not observe differences in a-WT animals, but found that the opposite situation occurs in a-KO mice, where transmission and LTP (but not LTD) seem to be favored in adulthood. These data suggest that Panx1 plays a pivotal role in the balance of plasticity mechanisms during aging. In this regard, it has been postulated that a shift in synaptic plasticity induction that promotes LTD over LTP during aging results from changes in expression, subunit composition and posttranslational modification of NMDAR, causing alterations in calcium influx (Foster, 1999; Kumar, 2011; Magnusson, 2012). Mounting evidence has shown differential roles of GluN2 subunits in LTP and LTD induction. For instance, early reports found that GluN2A/B antagonists block LTP, whereas GluN2C/D antagonists block LTD (Hrabetova and Sacktor, 1997; Hrabetova et al., 2000). More recent studies have shown that the selective blockade of GluN2B-containing NMDAR abolishes the induction of LTD, but not of LTP, whereas the preferential inhibition of GluN2A subunits prevents LTP without affecting LTD (Liu et al., 2004). On the other hand, the overexpression of GluN2B facilitates the synaptic potentiation induced by stimulation within the 10–100 Hz range (Tang et al., 1999), whilst the overexpression of GluN2A abolishes the induction of synaptic depression in the 3–5 Hz range (Cui et al., 2013). In addition, age-dependent differences in the expression of GluN2 subunits can also contribute to differences in the LTP and LTD induction observed during aging (Berberich et al., 2005; Bartlett et al., 2007), as GluN2 subunit expression is critically modified during development (Hestrin, 1992; Monyer et al., 1994; Sans et al., 2000). In this sense, we can speculate that the effects of the blockade or absence of Panx1 observed in our data could change the subunit composition of NMDARs or its active state (Figure 6). Similar shifts in the sliding modification threshold have been observed in transgenic mice overexpressing the GluN2B subunit of NMDAR (Tang et al., 1999) and in mice lacking calcineurin, which is a phosphatase that is crucial for the LTD mechanism (Zeng et al., 2001), suggesting a contribution of these factors to the effects observed when Panx1 is absent or inhibited. The observation that NMDA application induces LTD in both adult WT and KO mice suggests that the machinery for the expression of LTD is intact, but the signal to trigger this form of synaptic plasticity could be altered. These observations raised the question of how the blockade or absence of Panx1 modifies the synaptic activation of NMDAR and how this activation leads to an increase in LTP in detriment of LTD. Prochnow et al. (2012) suggest that the effects that they observed in excitability and LTP could be explained by a reduction in the catabolism of ATP released by Panx1, leading to a depletion of adenosine in the synaptic cleft and therefore facilitating the activation of NMDARs (Figure 6). Moreover, a recent report by Vroman et al. (2014), shows that extracellular ATP hydrolysis inhibits retinal synaptic transmission between horizontal cells and photoreceptors, by changing pH in synaptic cleft. ATP released by Panx1 channels located on horizontal cell dendrites, is hydrolyzed by ecto-ATPase into inosine, phosphate and protons ions acidifying the synaptic cleft. This change in the pH inhibits voltage dependent calcium channels present in the cone synaptic terminal, causing reduction in glutamate release by the cones. On the contrary, the closing of Panx1 channels prevents the release of ATP, and therefore stops the production of phosphate buffer and leads to alkalization of the synaptic cleft. A similar mechanism could occur in hippocampal Sc-CA1 synapse, since the opening of voltage gated calcium channels (VGCC) depends on both intracellular and extracellular pH (Tombaugh and Somjen, 1997) and hippocampus synaptic membranes present strong ecto-ATPase and ecto-ATPDase activity (Kukulski et al., 2004). Additionally, NMDARs also are modulated by extracellular pH (Gottfried and Chesler, 1994), where an increase in extracellular pH facilitates the activation, whereas a decrease in extracellular pH inhibits NMDAR. Thus, when Panx1 channels are blocked, a decrease in ATP release and the resulting alkalization could enhance synaptic strength (Figure 6). We believe that additional mechanisms could take place (Figure 6). One possible explanation is that the calcium concentration reached during LFS protocols through NMDARs in KO mice is greater than that in WT mice, enhancing kynase activation over phosphatase activation; nonetheless additional experiments are necessary to test this hypothesis. Regardless of the precise mechanism, the present findings emphasize a new role of Panx1 in excitatory hippocampal synaptic plasticity, where simply blocking Panx1 shifts the threshold balance between LTP and LTD. We have studied the effects of Panx1 on bidirectional synaptic plasticity in the Sc-CA1 pathway, because it is the most comprehensive synaptic model for NMDAR-dependent plasticity (Lüscher and Malenka, 2012). Similar synaptic plasticity processes have been described in other brain areas, as in neocortical and cerebellar synapses (Malenka and Bear, 2004). However, whether Panx1 is important to these brain areas remains to be elucidated.

## Conflict of interest statement

The authors declare that the research was conducted in the absence of any commercial or financial relationships that could be construed as a potential conflict of interest.

## References

[B1] Abbracchio M. P. Burnstock G. Verkhratsky A. Zimmermann H. (2009). Purinergic signalling in the nervous system: an overview. Trends Neurosci. 32, 19–29 10.1016/j.tins.2008.10.001 19008000

[B2] Anselmi F. Hernandez V. H. Crispino G. Seydel A. Ortolano S. Roper S. D. (2008). ATP release through connexin hemichannels and gap junction transfer of second messengers propagate Ca2+ signals across the inner ear. Proc. Natl. Acad. Sci. U S A 105, 18770–18775 10.1073/pnas.0800793105 19047635 PMC2596208

[B3] Ardiles A. O. Tapia-Rojas C. C. Mandal M. Alexandre F. Kirkwood A. Inestrosa N. C. (2012). Post-synaptic dysfunction is associated with spatial and object recognition memory loss in a natural model of Alzheimer’s disease. Proc. Natl. Acad. Sci. U S A 109, 13835–13840 10.1073/pnas.1201209109 22869717 PMC3427050

[B4] Bao L. Locovei S. Dahl G. (2004). Pannexin membrane channels are mechanosensitive conduits for ATP. FEBS Lett. 572, 65–68 10.1016/j.febslet.2004.07.009 15304325

[B5] Bartlett T. E. Bannister N. J. Collett V. J. Dargan S. L. Massey P. V. Bortolotto Z. A. (2007). Differential roles of NR2A and NR2B-containing NMDA receptors in LTP and LTD in the CA1 region of two-week old rat hippocampus. Neuropharmacology 52, 60–70 10.1016/j.neuropharm.2006.07.013 16904707

[B6] Berberich S. Punnakkal P. Jensen V. Pawlak V. Seeburg P. H. Hvalby Ø. (2005). Lack of NMDA receptor subtype selectivity for hippocampal long-term potentiation. J. Neurosci. 25, 6907–6910 10.1523/jneurosci.1905-05.2005 16033900 PMC6725356

[B7] Bliss T. V. Collingridge G. L. (1993). A synaptic model of memory: long-term potentiation in the hippocampus. Nature 361, 31–39 10.1038/361031a0 8421494

[B8] Bruzzone R. Hormuzdi S. G. Barbe M. T. Herb A. Monyer H. (2003). Pannexins, a family of gap junction proteins expressed in brain. Proc. Natl. Acad. Sci. U S A 100, 13644–13649 10.1073/pnas.2233464100 14597722 PMC263867

[B9] Collo G. North R. A. Kawashima E. Merlo-Pich E. Neidhart S. Surprenant A. (1996). Cloning OF P2X5 and P2X6 receptors and the distribution and properties of an extended family of ATP-gated ion channels. J. Neurosci. 16, 2495–2507 8786426 10.1523/JNEUROSCI.16-08-02495.1996 PMC6578782

[B10] Cone A. C. Ambrosi C. Scemes E. Martone M. E. Sosonsky G. E. (2013). A comparative antibody analysis of Pannexin1 expression in four rat brain regions reveals varying subcellular localizations. Front. Pharmacol. 4:6 10.3389/fphar.2013.00006 23390418 PMC3565217

[B11] Cui Z. Feng R. Jacobs S. Duan Y. Wang H. Cao X. (2013). Increased NR2A:NR2B ratio compresses long-term depression range and constrains long-term memory. Sci. Rep. 3:1036 10.1038/srep01036 23301157 PMC3539144

[B12] Dermietzel R. Traub O. Hwang T. K. Beyer E. Bennett M. V. Spray D. C. (1989). Differential expression of three gap junction proteins in developing and mature brain tissues. Proc. Natl. Acad. Sci. U S A 86, 10148–10152 10.1073/pnas.86.24.10148 2557621 PMC298664

[B13] Dudek S. M. Bear M. F. (1992). Homosynaptic long-term depression in area CA1 of hippocampus and effects of N-methyl-D-aspartate receptor blockade. Proc. Natl. Acad. Sci. U S A 89, 4363–4367 10.1073/pnas.89.10.4363 1350090 PMC49082

[B14] Dudek S. M. Bear M. F. (1993). Bidirectional long-term modification of synaptic effectiveness in the adult and immature hippocampus. J. Neurosci. 13, 2910–2918 8331379 10.1523/JNEUROSCI.13-07-02910.1993 PMC6576673

[B15] Fields R. D. Stevens B. (2000). ATP: an extracellular signaling molecule between neurons and glia. Trends Neurosci. 23, 625–633 10.1016/s0166-2236(00)01674-x 11137153

[B16] Foster T. C. (1999). Involvement of hippocampal synaptic plasticity in age-related memory decline. Brain Res. Rev. 30, 236–249 10.1016/s0165-0173(99)00017-x 10567726

[B17] Fouquet C. Petit G. H. Auffret A. Gaillard E. Rovira C. Mariani J. (2011). Early detection of age-related memory deficits in individual mice. Neurobiol. Aging 32, 1881–1895 10.1016/j.neurobiolaging.2009.11.001 20004498

[B18] Gottfried J. A. Chesler M. (1994). Endogenous H+ modulation of NMDA receptor-mediated EPSCs revealed by carbonic anhydrase inhibition in rat hippocampus. J. Physiol. 478, 373–378 7525946 10.1113/jphysiol.1994.sp020258 PMC1155659

[B19] Hestrin S. (1992). Developmental regulation of NMDA receptor-mediated synaptic currents at a central synapse. Nature 357, 686–689 10.1038/357686a0 1377360

[B20] Hrabetova S. Sacktor T. C. (1997). Long-term potentiation and long-term depression are induced through pharmacologically distinct NMDA receptors. Neurosci. Lett. 226, 107–110 10.1016/s0304-3940(97)00252-8 9159501

[B21] Hrabetova S. Serrano P. Blace N. Tse H. W. Skifter D. A. Jane D. E. (2000). Distinct NMDA receptor subpopulations contribute to long-term potentiation and long-term depression induction. J. Neurosci. 20:RC81 10827202 10.1523/JNEUROSCI.20-12-j0002.2000 PMC6772441

[B22] Huang Y. Grinspan J. B. Abrams C. K. Scherer S. S. (2007). Pannexin1 is expressed by neurons and glia but does not form functional gap junctions. Glia 55, 46–56 10.1002/glia.20435 17009242

[B23] Kim J.-E. Kang T.-C. (2011). The P2X7 receptor-pannexin-1 complex decreases muscarinic acetylcholine receptor-mediated seizure susceptibility in mice. J. Clin. Invest. 121, 2037–2047 10.1172/JCI44818 21505260 PMC3083785

[B24] Kukulski F. Sévigny J. Komoszyński M. (2004). Comparative hydrolysis of extracellular adenine nucleotides and adenosine in synaptic membranes from porcine brain cortex, hippocampus, cerebellum and medulla oblongata. Brain Res. 1030, 49–56 10.1016/j.brainres.2004.09.044 15567336

[B25] Kumar A. (2011). Long-term potentiation at CA3-CA1 hippocampal synapses with special emphasis on aging, disease and stress. Front. Aging Neurosci. 3:7 10.3389/fnagi.2011.00007 21647396 PMC3102214

[B26] Lee H. K. Takamiya K. Han J.-S. Man H. Kim C.-H. Rumbaugh G. (2003). Phosphorylation of the AMPA receptor GluR1 subunit is required for synaptic plasticity and retention of spatial memory. Cell 112, 631–643 10.1016/s0092-8674(03)00122-3 12628184

[B27] Lisman J. (1989). A mechanism for the Hebb and the anti-Hebb processes underlying learning and memory. Proc. Natl. Acad. Sci. U S A 86, 9574–9578 10.1073/pnas.86.23.9574 2556718 PMC298540

[B28] Liu L. Wong T. P. Pozza M. F. Lingenhoehl K. Wang Y. Sheng M. (2004). Role of NMDA receptor subtypes in governing the direction of hippocampal synaptic plasticity. Science 304, 1021–1024 10.1126/science.1096615 15143284

[B29] Locovei S. Scemes E. Qiu F. Spray D. C. Dahl G. (2007). Pannexin1 is part of the pore forming unit of the P2X(7) receptor death complex. FEBS Lett. 581, 483–488 10.1016/j.febslet.2006.12.056 17240370 PMC1868681

[B30] Locovei S. Wang J. Dahl G. (2006). Activation of pannexin 1 channels by ATP through P2Y receptors and by cytoplasmic calcium. FEBS Lett. 580, 239–244 10.1016/j.febslet.2005.12.004 16364313

[B31] Lüscher C. Malenka R. C. (2012). NMDA receptor-dependent long-term potentiation and long-term depression (LTP/LTD). Cold Spring Harb. Perspect. Biol. 4:a005710 10.1101/cshperspect.a005710 22510460 PMC3367554

[B32] MacVicar B. A. Thompson R. J. (2010). Non-junction functions of pannexin-1 channels. Trends Neurosci. 33, 93–102 10.1016/j.tins.2009.11.007 20022389

[B33] Magnusson K. R. (2012). Aging of the NMDA receptor: from a mouse’s point of view. Future Neurol. 7, 627–637 10.2217/fnl.12.54 23316115 PMC3540203

[B34] Malenka R. C. Bear M. F. (2004). LTP and LTD: an embarrassment of riches. Neuron 44, 5–21 10.1016/j.neuron.2004.09.012 15450156

[B35] Monyer H. Burnashev N. Laurie D. J. Sakmann B. Seeburg P. H. (1994). Developmental and regional expression in the rat brain and functional properties of four NMDA receptors. Neuron 12, 529–540 10.1016/0896-6273(94)90210-0 7512349

[B36] Panchin Y. Kelmanson I. Matz M. Lukyanov K. Usman N. Lukyanov S. (2000). A ubiquitous family of putative gap junction molecules. Curr. Biol. 10, R473–R474 10.1016/s0960-9822(00)00576-5 10898987

[B37] Pelegrin P. Surprenant A. (2006). Pannexin-1 mediates large pore formation and interleukin-1 beta release by the ATP-gated P2X7 receptor. EMBO J. 25, 5071–5082 10.1038/sj.emboj.7601378 17036048 PMC1630421

[B38] Penuela S. Bhalla R. Gong X.-Q. Cowan K. N. Celetti S. J. Cowan B. J. (2007). Pannexin 1 and pannexin 3 are glycoproteins that exhibit many distinct characteristics from the connexin family of gap junction proteins. J. Cell Sci. 120, 3772–3783 10.1242/jcs.009514 17925379

[B39] Prochnow N. Abdulazim A. Kurtenbach S. Wildförster V. Dvoriantchikova G. Hanske J. (2012). Pannexin1 stabilizes synaptic plasticity and is needed for learning. PLoS One 7:e51767 10.1371/journal.pone.0051767 23284764 PMC3527502

[B40] Rex C. S. Kramár E. A. Colgin L. L. Lin B. Gall C. M. Lynch G. (2005). Long-term potentiation is impaired in middle-aged rats: regional specificity and reversal by adenosine receptor antagonists. J. Neurosci. 25, 5956–5966 10.1523/jneurosci.0880-05.2005 15976084 PMC6724797

[B41] Romanov R. A. Bystrova M. F. Rogachevskaya O. A. Sadovnikov V. B. Shestopalov V. I. Kolesnikov S. S. (2012). The ATP permeability of pannexin 1 channels in a heterologous system and in mammalian taste cells is dispensable. J. Cell Sci. 15, 5514–5523 10.1242/jcs.111062 22956545 PMC3561859

[B42] Sans N. Petralia R. S. Wang Y. X. Blahos J. 2nd Hell J. W. Wenthold R. J. (2000). A developmental change in NMDA receptor-associated proteins at hippocampal synapses. J. Neurosci. 20, 1260–1271 10648730 10.1523/JNEUROSCI.20-03-01260.2000 PMC6774158

[B43] Santiago M. F. Veliskova J. Patel N. K. Lutz S. E. Caille D. Charollais A. (2011). Targeting pannexin1 improves seizure outcome. PLoS One 6:e25178 10.1371/journal.pone.0025178 21949881 PMC3175002

[B44] Silverman W. R. de Rivero Vaccari J. P. Locovei S. Qiu F. Carlsson S. K. Scemes E. (2009). The pannexin 1 channel activates the inflammasome in neurons and astrocytes. J. Biol. Chem. 284, 18143–18151 10.1074/jbc.M109.004804 19416975 PMC2709345

[B45] Tang Y. P. Shimizu E. Dube G. R. Rampon C. Kerchner G. A. Zhuo M. (1999). Genetic enhancement of learning and memory in mice. Nature 401, 63–69 10.1038/43432 10485705

[B46] Thomas-Crusells J. Vieira A. Saarma M. Rivera C. (2003). A novel method for monitoring surface membrane trafficking on hippocampal acute slice preparation. J. Neurosci. Methods 23, 159–166 10.1016/s0165-0270(03)00050-5 12763242

[B47] Thompson R. J. Jackson M. F. Olah M. E. Rungta R. L. Hines D. J. Beazely M. A. (2008). Activation of pannexin-1 hemichannels augments aberrant bursting in the hippocampus. Science 322, 1555–1559 10.1126/science.1165209 19056988

[B48] Tombaugh G. C. Somjen G. C. (1997). Differential sensitivity to intracellular pH among high- and low- threshold Ca2+ currents in isolated rat CA1 neurons. J. Neurophysiol. 77, 639–653 9065837 10.1152/jn.1997.77.2.639

[B49] Vogt A. Hormuzdi S. G. Monyer H. (2005). Pannexin1 and Pannexin2 expression in the developing and mature rat brain. Brain Res. Mol. Brain Res. 141, 113–120 10.1016/j.molbrainres.2005.08.002 16143426

[B50] Vroman R. Klaassen L. J. Howlett M. H. Cenedese V. Klooster J. Sjoerdsma T. (2014). Extracellular ATP hydrolysis inhibits synaptic transmission by increasing ph buffering in the synaptic cleft. PLoS Biol. 12:e1001864 10.1371/journal.pbio.1001864 24844296 PMC4028192

[B51] Wang Y. Haughey N. J. Mattson M. P. Furukawa K. (2004). Dual effects of ATP on rat hippocampal synaptic plasticity. Neuroreport 15, 633–636 10.1097/00001756-200403220-00012 15094466

[B52] Yamazaki Y. Kaneko K. Fujii S. Kato H. Ito K. (2003). Long-term potentiation and long-term depression induced by local application of ATP to hippocampal CA1 neurons of the guinea pig. Hippocampus 13, 81–92 10.1002/hipo.7999 12625460

[B53] Zeng H. Chattarji S. Barbarosie M. Rondi-Reig L. Philpot B. D. Miyakawa T. (2001). Forebrain-specific calcineurin knockout selectively impairs bidirectional synaptic plasticity and working/episodic-like memory. Cell 107, 617–629 10.1016/s0092-8674(01)00585-2 11733061

[B54] Zoidl G. Petrasch-Parwez E. Ray A. Meier C. Bunse S. Habbes H. W. (2007). Localization of the pannexin1 protein at postsynaptic sites in the cerebral cortex and hippocampus. Neuroscience 146, 9–16 10.1016/j.neuroscience.2007.01.061 17379420

